# Differential Host Pro-Inflammatory Response to Mycobacterial Cell Wall Lipids Regulated by the *Mce1* Operon

**DOI:** 10.3389/fimmu.2020.01848

**Published:** 2020-08-18

**Authors:** Jéssica D. Petrilli, Igor Müller, Luana E. Araújo, Thiago M. Cardoso, Lucas P. Carvalho, Bruna C. Barros, Maurício Teixeira, Sérgio Arruda, Lee W. Riley, Adriano Queiroz

**Affiliations:** ^1^Laboratorio Avançado de Saúde Pública, Instituto Gonçalo Moniz, Salvador, Brazil; ^2^Laboratório de Pesquisa Clínica, Instituto Gonçalo Moniz, Salvador, Brazil; ^3^Division of Infectious Diseases and Vaccinology, School of Public Health, University of California, Berkeley, Berkeley, CA, United States

**Keywords:** *Mycobacterium tuberculosis*, lipid-induced responses, mce1 operon, mycobacterial cell wall lipid, inflammation

## Abstract

The cell wall of wild-type (WT) *Mycobacterium tuberculosis* (Mtb), an etiologic agent of tuberculosis (TB) and a Mtb strain disrupted in a 13-gene operon *mce1* (Δmce1) varies by more than 400 lipid species. Here, we examined Mtb lipid-induced response in murine macrophage, as well as in human T-cell subpopulations in order to gain an insight into how changes in cell wall lipid composition may modulate host immune response. Relative to WT Mtb cell wall lipids, the non-polar lipid extracts from Δmce1 enhanced the mRNA expression of lipid-sense nuclear receptors TR4 and PPAR-γ and dampened the macrophage expression of genes encoding TNF-α, IL-6, and IL-1β. Relative to untreated control, WT lipid-pre-stimulated macrophages from healthy individuals induced a higher level of CD4^−^CD8^−^ double negative T-cells (DN T-cells) producing TNF-α. Conversely, compared to WT, stimulation with Δmce1 lipids induced higher mean fluorescence intensity (MFI) in IL-10-producing DN T cells. Mononuclear cells from TB patients stimulated with WT Mtb lipids induced an increased production of TNF-α by CD8^+^ lymphocytes. Taken together, these observations suggest that changes in *mce1* operon expression during a course of infection may serve as a strategy by Mtb to evade the host pro-inflammatory responses.

## Introduction

Despite the availability of effective treatment regimens against *Mycobacterium tuberculosis* (Mtb), an etiologic agent of tuberculosis (TB), TB remains a major public health challenge, which has surpassed AIDS as the most common infectious disease cause of death in adults (1). A large proportion of the world's population latently infected with Mtb serves as the main reservoir of reactivation TB (2). The mechanisms by which Mtb remains persistent in a host and how a subset of the infected hosts reactivates to develop TB are still not fully understood.

Mtb contains four homologous copies of an operon called *mce*1-4, which resemble ATP-binding cassette transporters possibly involved in lipid important across its cell wall (3–5). Disruption of one of these operons (*mce1*) precludes Mtb to induce a strong Th1 type T-cell immune response and to form organized granuloma in mouse lungs (6). The expression of *mce1* operon is repressed in the WT strain during the first 8 weeks of mouse infection (7) and its expression decreases 4 h post infection in macrophages (8). Thus, at some point during *in vitro* macrophage or mouse infection, the WT strain displays the same phenotype shown by the *mce1* operon mutant (Δmce1).

The cell wall lipid content of Mtb is highly affected by the repression of the *mce1* operon. The mutant strain accumulates several-fold greater amount of free mycolic acids on its surface (9, 10). As revealed by lipid metabolomics analysis, the mutant also contains diminished amounts of diacyltrehalose, diacylated sulfoglycolipid, phosphatidylethanolamine, phthienoic acid, and phthioceranic acid in its cell wall compared to its parent wild-type (WT) strain. In fact, a lipidomics analysis has shown differential expression of more than 400 lipid species between the mutant and WT Mtb strains (11).

The cell wall lipid rearrangement during infection could be a determining factor for bacterial persistence, as previously suggested (12). Individually, such lipids have been shown to induce granuloma formation (13), leukocyte migration (14) and inflammatory cytokines expression (15), or to inhibit macrophage responses (16–18). However, the evaluation of host cell behavior in response to Mtb cell wall lipid rearrangement has never been assessed. Here, we hypothesized that by comparing responses of host immune cells challenged *in vitro* with lipids extracted from the *mce1* operon mutant or WT Mtb, we may be able to demonstrate how differences in cell wall lipid composition affect host cell responses that would be advantageous to Mtb.

## Materials and Methods

### Bacterial Strains, Media, and Growth Conditions

The following bacterial strains were used: *mce1* operon mutant *M. tuberculosis* (Δmce1) and its parent Erdman wild-type (WT) strain. The generation of Δmce1 was previously described by Shimono et al. (6). Both Mtb strains were initially cultured in Middlebrook 7H9 broth (Difco, MD) containing 10% ADC (Beckton-Dickinson, MD) and 0.2% glycerol (Fisher Scientific, NJ) to obtain similar numbers of bacterial cells for growth in detergent-free minimal media. Bacteria were incubated at 37°C until stationary phase in Sauton's media (without Tween) as previously described (19). Briefly, bacteria were grown in 125 mL polycarbonate bottles containing 30 mL of Sauton's media with 300 μl of saturated planktonic culture (OD of each test strain was adjusted so that equal numbers of each bacteria strain were inoculated) and incubated without agitation at 37°C for 19 days.

### Lipid Extraction

The extraction of lipids from biofilm cultures was performed as previously described (19). Briefly, biofilm from 19-day *M. tuberculosis* cultures was harvested, and apolar lipids were extracted from using 5 mL of methanol: 0.3% NaCl (100:10) mixed with 2.5 mL of petroleum ether, incubated at room temperature for 30 min. The upper petroleum ether layer containing the apolar lipids was separated by centrifugation. After solvent evaporation, apolar lipids were weighed and resuspended at a concentration of 0.02 mg/mL in hexane/isopropanol. Lipid extracts (0.5 mL) were layered onto 24-well tissue culture plates (0.01 mg/well) and the solvent was allowed to evaporate. Control wells were layered only with hexane/isopropanol in the absence of lipid extracts.

### RAW Macrophage Assay

#### RAW 264.7 Murine Macrophage Cultures

RAW 264.7 murine macrophage-like cells (ATCC TIB-71) were cultured and maintained in Dulbecco's modified Eagles medium (DMEM; Gibco) supplemented with 10% FBS at 37°C, 5% CO_2_, in a humidified incubator. Macrophage cell number and viability were assessed by staining in a trypan blue (Gibco) exclusion assay. Prior to experimentation, macrophages were first seeded in 75-cm^2^ flasks overnight to achieve 70% confluency, and then seeded onto Mtb lipid-coated 24-well tissue culture plates at a concentration of 3.7 x 10^5^ cells/well, and incubated at 37°C in a 5% CO_2_ humidified atmosphere for 72 h. Control macrophages were cultured on layered wells with hexane/isopropanol, and incubated in the absence of lipid extracts.

#### RNA Extraction and Purification

RNA was extracted from RAW cells according to a standard Trizol RNA extraction protocol supplied by Invitrogen (Invitrogen, Life Technologies). Extracted RNA was treated with DNase (Qiagen) to ensure that no DNA was present in the samples. DNA-free RNA (500 ng) was mixed with 50 ng of random hexamers and 50 μM of oligo (dT) (Invitrogen) at a final volume of 10 μl, then reverse transcribed to cDNA with Superscript III reverse transcriptase (Invitrogen) following the manufacturer's recommendations.

#### qPCR

Nine genes encoding lipid-sensing nuclear receptors (LSNR) were selected as targets, in addition to genes related to pro-inflammatory response (complete list of genes available in Supplementary Table 1). Primers were designed to produce a 100–195 bp amplicon for each gene. qPCR reactions were performed using 25 ng of previously generated cDNA and Maxima SYBR Green/ROX qPCR Master Mix (2X) (Fermentas) in accordance with the manufacturer's instructions. Relative changes in gene expression between lipid-induced and unstimulated RAW cells (controls) were analyzed by 2^−ΔΔ*CT*^ method according to a previously described method (20). The expression of all tested genes was normalized to both β*-actin* and *glyceraldehyde 3-phosphate dehydrogenase* (GAPDH) gene expression.

### Assays With Peripheral Blood Mononuclear Cells Isolated From Healthy Subjects and TB Patients

#### Study Participants

Seventeen participants were enrolled in this study, divided into healthy subjects (*n* = 11) and TB patients (*n* = 6). All healthy volunteers, recruited from the Gonçalo Moniz Institute (IGM-Fiocruz), were tested for latent TB infection (LTBI) by interferon γ release assay (QuatiFERON® TB Gold); two of them were tested positive for LTBI. Six TB patients were recruited at the Octavio Mangabeira Hospital (HEOM), a reference center for respiratory disease in Salvador, Bahia-Brazil. All patients were diagnosed with active TB based on GeneXpert MTB/RIF test results. The age of the study subjects ranged between 18 and 60 years and all participants were HIV negative.

#### PBMC Isolation From Study Subjects and Culture Conditions

Peripheral blood mononuclear cells (PBMC) were obtained by the Ficoll-Paque (GE Healthcare) density gradient method. PBMC concentrations were adjusted to 1 x 10^6^ cells/mL in 1 mL of complete RPMI 1640 (100 μl/mL gentamicin, 2 mM L-glutamine, 30 mM HEPES) containing 10% inactivated bovine fetal serum (FBS) (Life technologies GIBCO BRL, Gaithersburg, MD). PBMCs from TB patients were cryopreserved (FBS containing 10% DMSO) in liquid nitrogen prior to performing culture assays. All PBMCs were dispensed into previously prepared 24-well plates and incubated at 37°C under 5% CO_2_.

#### Co-culturing of Adherent and Non-adherent Cells

Adherent cells were isolated from six healthy subjects PBMC (2 × 10^7^) on sterile 6-well tissue culture plates. After 2 h of adherence, cells in suspension (non-adherent cells) were harvested and cryopreserved (FBS with 10% DMSO) at −80°C. Adherent cells were kept on 6-well plates for 5 days at 37°C under 5% CO_2_. After differentiation, macrophages were collected by washing the wells with 2 mM EDTA saline at 4°C, with the aid of a cell-scraper. Cells (1 × 10^6^) were then cultured on 24-well tissue culture plates in RPMI medium supplemented with 10% FBS in the presence or absence of Mtb non-polar lipids for 72 h at 37°C under 5% CO_2_. Non-adherent cryopreserved cells were then added to pre-stimulated macrophage cultures at a ratio of 10:1 (non-adherent cells:macrophage), incubated for 72 h at 37°C under 5% CO_2_, and finally stained to perform flow cytometry.

#### PBMC Assay With TB Patients

Cryopreserved PBMCs from TB patients (1 × 10^6^) were thawed and incubated for 72 h on 24-well tissue culture plates pre-treated with lipids at 37°C under 5% CO_2_ in the presence or absence of Mtb non-polar lipids (0.01 mg/well) extracted from WT and Δmce1 strains. After incubation, cells were stained for flow cytometry analysis.

#### Cytokine Analysis

Supernatants of PBMCs from healthy subjects and TB patients were collected and stored at −20°C for cytokine analysis (TNF-α, IL-6, IL-1β, IFN-γ, and IL-10) by either ELISA (R&D Systems, Minneapolis, MN) or Cytometric Bead Array (BD™ CBA Human TH1/TH2 cytokine kit) according to the manufacturer's instructions. Results are expressed as pg/mL.

#### Flow Cytometry

Co-cultured cells were stained with CD3-FITC (clone SP34-2), CD4-APC-CY7 (clone RPA-T4), and CD8-PerceP-CY5.5 (clone RPA-T8). PBMCs from TB patients were stained with CD3-APC-CY7 (clone HIT3a), CD4-APC (clone RPA-T4), CD8-ALEXA700 (clone 53-6.7), CD56-Brilliant Violet 421 (clone HCD56), CD38-PE-CY5 (clone HIT2), and CD14-FITC (clone 61D3). All cells were then fixed with 4% PFA (BD Cytofix/Cytoperm Fixation/Permeabilization Solution Kit with GolgiPlug™). For intracellular staining, fixed cells were permeabilized using a cytofix/cytoperm kit (BD Biosciences) and stained intracellularly with anti-TNF, IFN-γ, and IL-10 antibodies. Cells were acquired (100,000 events) on a BD LSRFortessa® device. The frequencies of stained cells and MFI were estimated using FlowJo 7.10.1 software (Tree Star, Inc, Ashland, OR.).

### Statistical Analysis and Data Representation

Statistical analyses were performed with GraphPad Prism v.7.0 (GraphPad Inc., San Diego, CA, USA). Statistical significance between variables was assessed by Mann-Whitney U test or Friedman test, followed by Dunn's post-test. Continuous variables with normal distribution were expressed as mean ± standard deviation, while those with non-normal distributions were expressed as median and interquartile interval. Significance was considered when *p* < 0.05.

## Results

### Δmce1 Lipid Extracts Dampen the Pro-inflammatory Response in Murine Macrophages

**Figure 1:**
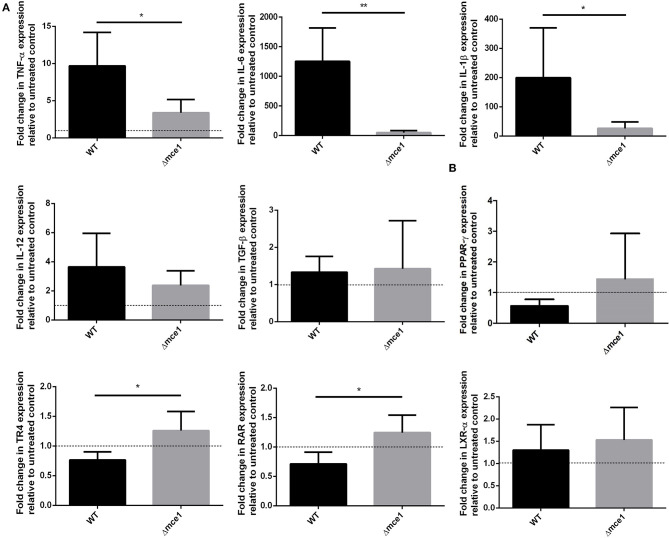
qPCR analysis of selected cytokine genes **(A)** and lipid-sense nuclear receptor (LSNR) genes **(B)** in murine macrophages exposed to Mtb apolar lipid extracts. Data are presented as fold-difference in gene expression levels in cells stimulated with apolar lipids extracted from wild type or *mce1* operon mutant *M. tuberculosis*, relative to untreated control, mean ± SD; gene expression was normalized to β-actin and glyceraldehyde 3-phosphate dehydrogenase (GAPDH) genes; six replicates of each reaction were performed. Statistical significance was evaluated by Mann-Whitney *U* test. *p* < 0.05. *p < 0.05; **p < 0.01. WT, *Mycobacterium tuberculosis* wild type strain; Δmce1, strain with disruption of *mce1* operon.

The transcriptional expression of the pro-inflammatory genes encoding *TNF-*α, *IL-6*, and *IL-1*β in RAW cells, stimulated by either Δmce1 or WT lipid extracts, was evaluated (Figure 1A). The expression of these genes was, respectively, 9.7-, 1,250-, and 199-fold higher in macrophages stimulated with WT lipid extract than in untreated control cells. Conversely, Δmce1 lipid stimulation dampened the expression of *TNF-*α, *IL-6*, and *IL-1*β genes by 3.4-, 47-, and 26-fold, respectively. The WT/Δmce1 expression ratios of these markers were, respectively, 2.8, 27, and 7.6 (*p* < 0.05, 0.01, and 0.05). *IL-12* was increased 3.6- and 2.4-fold in macrophages stimulated by WT and Δmce1 lipids, respectively (raw data in Supplementary Table 2).

### Differential Induction of Lipid Sense Nuclear Receptors (LSNR) by Mtb Lipids

Mtb interacts with LSNR to modulate macrophage immune and metabolic functions (21, 22). Since mycobacterial lipid extracts modulated pro-inflammatory response in macrophages, we attempted to determine whether LSNR activation was involved in this process. Here, we studied the expression by macrophages of LSNR PPAR-γ, TR4, RAR, and LXR-α genes. As depicted in Figure 1B, while the WT lipid extracts consistently repressed *PPAR-*γ, *TR4*, and *RAR* expression by 0.6-, 0.8-, and 0.7-fold, respectively, the lipid extracts of the mutant strain enhanced the expression of these same genes by 1.4-, 1.2-, and 1.2-fold, respectively, relative to untreated controls. The resulting Δmce1/WT expression ratios were, respectively, 2.3, 1.5, and 1.7. Fold-differences between groups were statically significant for *TR4* and *RAR* (*p* < 0.05). Both lipid extracts, WT and Δmce1, induced similar expression levels of another LSNR gene, *LXR-*α (1.3- and 1.5-fold, respectively).

### Macrophages Pre-stimulated by Mycobacterial Lipids Activate Both CD4^+^ and Double-Negative (CD4^−^ CD8^−^) T Cells

A co-culture assay was performed with both non-adherent and lipid-pre-treated adherent cells isolated from the peripheral blood of healthy subjects. Following the activation of IFN-γ-, TNF-α-, and IL-10-producing CD4^+^ and CD8^+^ T cells by lipid-stimulated macrophages, the proportion of IL-10-producing CD4^+^ was found to be higher in Δmce1 lipid-induced cells than in those stimulated by WT (*p* < 0.05) (Supplementary Figure 1A). Also, compared to untreated control, stimulation with Δmce1 lipids induced lower mean fluorescent intensity (MFI) in TNF-α-producing CD4^+^ T cells (*p* < 0.05) (Supplementary Figure 1A). However, neither the frequency nor the MFI of CD8^+^ T cells changed (Supplementary Figure 1B).

**Figure 2:**
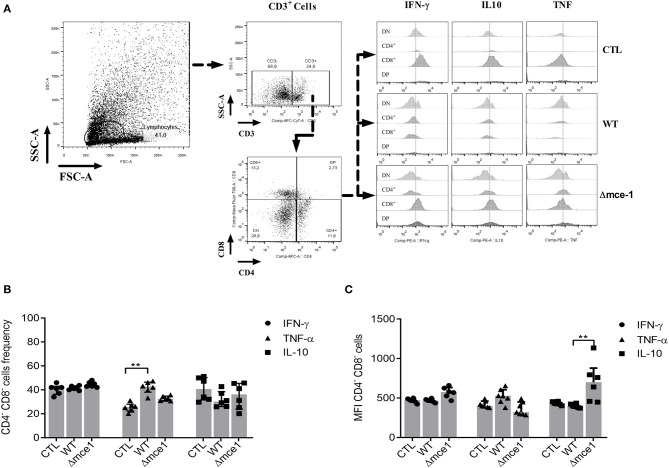
IFN-γ-, TNFα- and IL-10-producing by CD4^−^CD8^−^ double negative (DN) T cells from six healthy donors in a co-culture assay. Non-adherent cells were added to a culture containing adherent cells exposed to apolar lipids extracted from wild type vs *mce1* operon mutant *M. tuberculosis*. **(A)** The gating strategy to assess the DN T cells and cytokine production by each cell population. **(B)** Frequency of IFN-γ-, TNFα-, and IL-10 produced by DN T cells. **(C)** Quantification of the mean fluorescence intensity of IFN-γ-, TNFα-, and IL-10-producing DN T cells. Results are expressed as median ± IQR; *n* = 6/group. Statistical significance was determined by Friedman test followed by Dunn's post-test; significance was considered at **p < 0.01. WT, Mtb wild type strain; Δmce1, Mtb strain disrupted in *mce1 operon*; CTL, no stimulus.

The co-culture assay showed that pre-stimulation with both WT and Δmce1 lipids also increase the frequency and MFI of co-cultured DN T cells (Figure 2), which were defined as CD3 positive but CD4 and CD8 negative (CD3^+^CD4^−^CD8^−^) (Figure 2A). The proportion of TNF-α-producing DN T cells was only found to be higher in WT lipid-induced cells than in non-stimulated controls (*p* ≤ 0.01) (Figure 2B). Interestingly, compared to WT, stimulation with Δmce1 lipids induced higher MFI in IL-10-producing DN T cells (*p* ≤ 0.01) (Figure 2C).

### Cytokine Production in Cultures of Lipid-Induced PBMC

**Figure 3:**
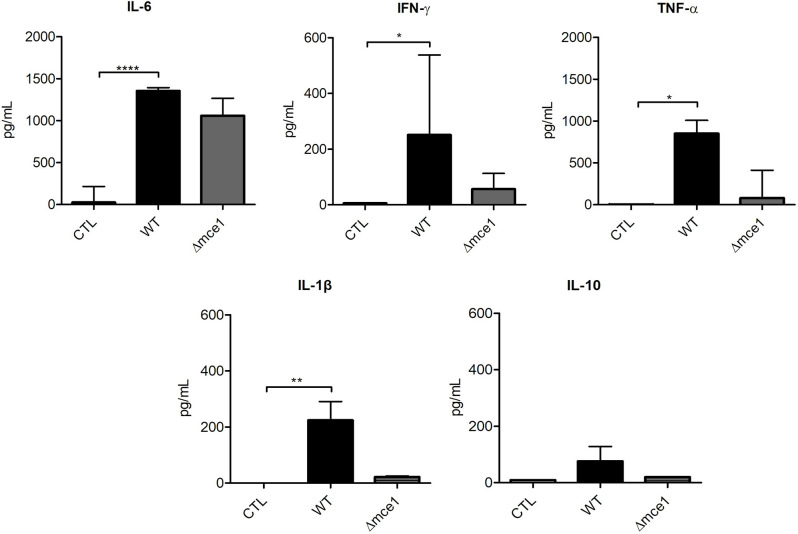
Cytokine production in peripheral blood mononuclear cells (PBMC) from six healthy donors exposed to apolar lipids extracted from wild type vs *mce1* operon mutant *M. tuberculosis*. Results are expressed as median ± IQR; *n* = 6/group. Statistical significance was determined by Friedman test followed by Dunn's post-test; significance was considered at *p < 0.05; **p < 0.01; ****p < 0.0001. WT, Mtb wild type strain; Δmce1, Mtb strain disrupted in *mce1 operon*; CTL, no stimulus.

The levels of cytokines IL-6, IFN-γ, TNF-α, IL-1β, and IL-10 were assessed in the supernatants of PBMCs stimulated with mycobacterial lipids (Figure 3). As expected, cells stimulated with WT lipids showed increased levels of pro-inflammatory cytokines IL-6 (1,353 pg/mL), IFN-γ (317 pg/mL), TNF-α (851 pg/mL) and IL-1β (224 pg/mL) relative to non-stimulated controls (26, 5, 4.5, and 0 pg/mL, respectively).

### Characterization of Lipid-Induced Response in PBMCs Isolated From Patients With Active TB

**Figure 4:**
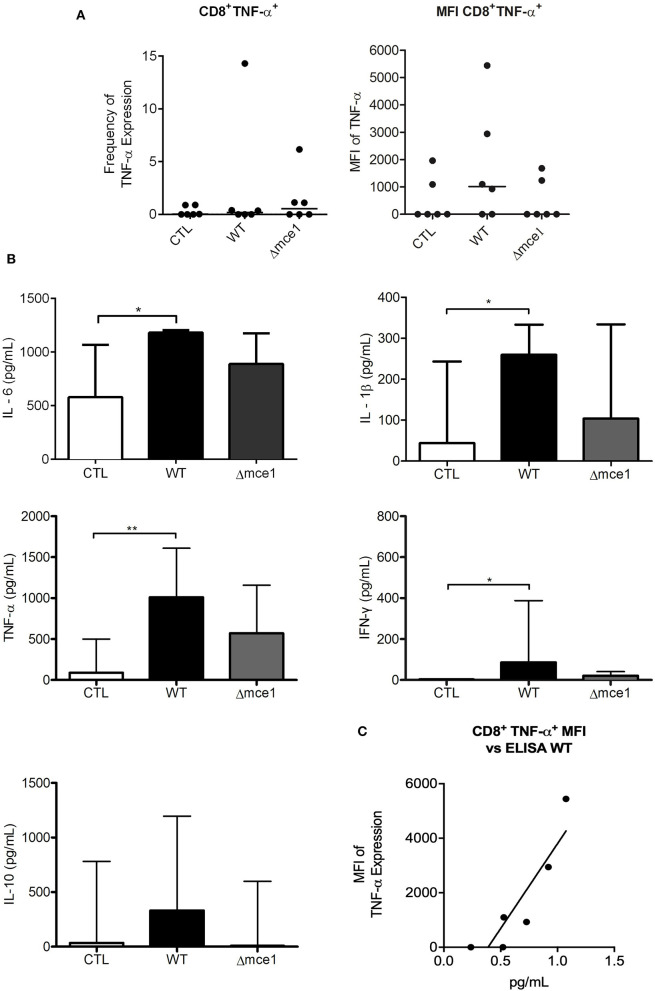
Peripheral blood mononuclear cell (PBMC) and cytokine production from six TB patients exposed to apolar lipids extracted from wild type vs *mce1* operon mutant *M. tuberculosis*. **(A)** Frequency and quantification of the mean fluorescence intensity of CD8^+^ T lymphocyte. **(B)** Cytokines production from PBMC cultures. **(C)** Analysis of correlation between the expression of TNF-α in CD8^+^ T cell and cytokine release in PBMC supernatant. Results are expressed as median ± IQR; *n* = 6/group Statistical significance was determined by Friedman test followed by Dunn's post-test; significance was considered at *p < 0.05; **p < 0.01. WT, Mtb wild type strain; Δmce1, Mtb strain disrupted in *mce1 operon*; CTL, no stimulus.

Next, to evaluate the effects of stimulation with WT and Δmce1 lipid extracts on the inflammatory response in PBMCs isolated from patients with active TB (Figure 4), the frequency of CD4^+^, CD8^+^, CD56^+^, NKT, CD4^−^/CD8^−^ DN, CD38^+^, and CD14^+^ subpopulations were assessed, in addition to the production of IFN-γ, TNF-α, and IL-10 by these cells (see Supplementary Figure 2A). Compared to untreated control, stimulation with Δmce1 lipids induced lower MFI in IFN-γ-producing DN T cells (*p* ≤ 0.05) (Supplementary Figure 2B). In contrast to the response seen in DN T cells from healthy subjects stimulated with Mtb lipids, no significant differences were observed in the Mtb lipid-stimulated TNF-α- and IL-10-producing DN T cells from patients with active TB compared to untreated cells.

The proportion of IFN-γ-producing CD14^+^ cells stimulated by Δmce1 lipids was higher than that in WT lipid-induced cell cultures (*p* < 0.05) (Supplementary Figure 2B). Interestingly, although not statistically significant, the MFI of CD8^+^ cells-producing TNF-α increased 3.4-fold in WT lipid-stimulated PBMC cultures relative to untreated controls (*p* > 0.05) (Figure 4A). The levels of IL-6, IL-1β, TNF-α, and IFN-γ were increased in WT lipid-induced PBMCs relative to untreated controls (*p* < 0.05) (Figure 4B). Importantly, the production of TNF-α seen in supernatant of WT lipid-stimulated PBMCs was strongly correlated to MFI of CD8^+^ T cells-producing TNF-α (*p* < 0.05; Pearson *r* = 0.8) (Figure 4C).

## Discussion

Mice infected with a strain of *M. tuberculosis* disrupted in the *mce1* operon are unable to mount a strong pro-inflammatory response against WT Mtb, as evidenced by decreased levels of IL-6 and TNF-α; consequently, these animals do not form organized granulomas (6). Recent studies have demonstrated that free mycolic acid accumulates on the surface of Mtb mutant cells (9, 11), and that the cell wall contains decreased levels of some saccharolipids and glycerophospholipids species. Only residual or no alteration were observed in the complemented strain, relative to WT (11). As the *mce1* operon of WT Mtb becomes repressed during the course of infection in a mouse model of TB (7), we reasoned that the lipids in the cell wall of this *mce1* mutant could be representative of the lipid composition of WT Mtb at some point during a course of *in vivo* infection. Thus, we hypothesized that the Mce1-mediated remodeling of lipids in the Mtb cell wall may contribute to the differential host pro-inflammatory response that could facilitate the persistence of Mtb in the infected host.

When stimulated with WT or Δmce1 lipid extracts, the RAW cells evaluated herein exhibited opposite expression responses in genes encoding *TNF-*α, *IL-6* and *IL-1*β (Figure 1A), as lipids extracted from the *mce1* operon mutant strain were found to virtually reverse the pro-inflammatory response triggered by stimulation with WT lipids. One mechanism for this modulation of inflammatory response may involve the mutant's lipids interacting with LSNRs PPAR-γ, TR4 and RAR (Figure 1B). Mtb lipids can serve as ligands of LSNRs, a large family of intracellular proteins responsible for regulating gene transcription (23). Previous studies have shown that Mtb infection is associated with increased expression of PPAR-γ and TR4 leading to Mtb survival through IL-10 production and prevention of phagolysosome maturation (21, 22). Also, PPAR-γ ligands inhibit monocyte production of TNF-α, IL-6, and IL-1β (24) and the transcriptional activation of the pro-inflammatory transcription factor NF-κB in murine macrophage cells (25). Also, TR4 is sensed by mycolic acids (26), which is greatly increased in amount in the cell wall of the mutant strain (9, 12).

On the other hand, we found that lipid extracts from both Mtb strains induced a similar expression level (1.3- and 1.5-fold) of another LSNR LXR-α (Figure 1B). This transcriptional factor triggers a protective immune response against Mtb (22, 27) and it is activated by oxysterols, the oxygenated derivatives of cholesterol (28, 29). As far as known, oxysterols are not included among those mycobacterial cell wall lipids that are altered by Mce1 repression (11), which may explain why we did not see a difference in the expression of LXR-α by cells exposed to lipids from the WT or mutant Mtb (30).

**Figure 5:**
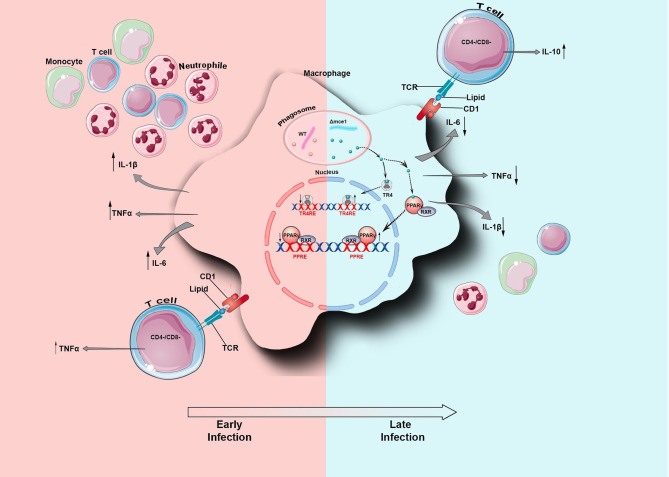
Schematic representation of *Mycobacterium tuberculosis* (Mtb) lipid-induced host inflammatory adaptation in macrophage. During infection and 4 h after phagocytosis by the macrophage, the *mce1* operon of Mtb is naturally repressed (8) and the bacterial cell undergoes cell wall lipid rearrangement (12). At the early phase of infection, the WT lipids induce the macrophage to secrete TNF-α, IL-1β, and IL-6. Also, these lipids are presented by CD1 to TNF-α-producing CD4^−^CD8^−^ double negative (DN) T cells. Together, these secreted cytokines should recruit immune cells to the site of infection. Conversely, during the later phase of infection, the Δmce1 lipids interact with the LSNR PPAR-γ and TR4, which in turn may negatively modulate the secretion of pro-inflammatory cytokines. These lipids are also presented by CD1 to IL-10- and IFN-γ-producing DN T cells. WT, Mtb wild type strain; Δmce1, Mtb strain disrupted in *mce1 operon*; TR4, testicular nuclear receptor 4; PPAR-γ, Peroxisome proliferator-activated receptor gamma.

Macrophages can present mycobacterial lipids via the CD1 (CD1a–CD1e) family of MHC-class-I-like glycoproteins to T cells (27). Depending on their species, these lipids can be presented by CD1a, CD1b, or CD1c to either: (A) CD4^−^CD8^−^ double negative or to CD4^+^ and CD8^+^ αβ TCR clonally diverse T-cells that mediate adaptive immunity, or (B) be presented by CD1d molecules to natural killer T (NKT) cells (15, 31). Thus, since the lipid rearrangement on Mtb cell wall dampens the macrophage inflammatory response, we wondered if this modulation would determine the pattern of T cell activation. Here, compared to WT, Δmce1 lipid-pre-stimulated macrophages induced higher frequency and MFI level of, respectively, IL-10-producing CD4^+^ and DN T cells (Supplementary Figure 1 and Figure 2). Conversely, relative to untreated control, WT lipids induced a higher proportion of TNF-α-producing DN T-cells and Δmce1 lipids decreased the MFI level in TNF-α-producing CD4^+^ T-cells. CD8^+^ T-cells, isolated from healthy subjects, were not activated by lipid-pre-stimulated macrophages (Supplementary Figure 1). Taken together, these results suggest that the Mtb's cell wall lipid-rearrangement modulates the macrophage's inflammatory response and, indirectly, determine the function of both CD4^+^ and DN T-cells. In Figure 5, we propose a model of *mce1* operon-mediated differential Mtb lipid-induced host inflammatory control in macrophage.

We have not determined which DN T-cell population subsets respond to each lipid extract used here, but we can speculate that WT lipids would induce the production of TNF-α through αβ DN T-cells, while the Δmce1 lipids may activate the IL-10-producing γδ DN T-cells by lymphocyte-dependent immunoregulatory mechanisms, as previously suggested (32).

In contrast to the results with healthy donors, in PBMC collected from TB patients, the MFI of TNF-α-producing CD8^+^ T-cells induced by WT lipids was 3-fold higher than in non-stimulated or Δmce1 lipids-stimulated PBMC, suggesting distinct roles of Mtb lipids in activating T lymphocytes subpopulations in healthy and TB subjects. The TNF-α production by CD8^+^ T-cells is supported by the increased level of this cytokine in culture supernatants of WT lipid-induced PBMC relative to untreated control, suggesting that CD8^+^ T-cells stimulated by mycobacterial lipids maybe an important source of TNF-α during active disease.

*In vivo* studies showed that the expression of *mce1* operon is repressed in the WT strain during the infection in both mouse (7) and macrophages (8) due the overexpression of mce1R gene. Interestingly, previous *in vitro* studies showed that under a hypoxic growth condition, the level of cell wall free mycolic acids in Mtb increases while the levels of trehalose mono (TMM) and dimycolate (TDM) decrease and re-aeration reverses such changes (33, 34). TDM is widely known as cord factor and serve as potent triggers of host inflammation (35). Taken together, we can speculate that the hypoxic environment in tuberculosis granuloma may trigger a Mtb cell wall lipid remodeling via repression of *mce1* operon. LSNR may serve as one component of this process to negatively dampen macrophage pro-inflammatory response, and perhaps indirectly influence T-cell-dependent pro-inflammatory responses. This lipid-mediated microbial defense strategy may contribute to Mtb's long-term survival in an infected host.

## Data Availability Statement

The datasets generated for this study are available on request to the corresponding author.

## Ethics Statement

The studies involving human participants were reviewed and approved by Gonçalo Moniz Institute (IGM-Fiocruz, Bahia) CAAE: 76009417.9.0000.0040. The patients/participants provided their written informed consent to participate in this study.

## Author Contributions

JP, LC, and AQ made substantial contributions to conception and design. JP, TC, BB, and MT made substantial contributions to acquisition of data and analysis and interpretation of data. SA and LR participated in drafting the article or revising it critically for important intellectual content, and AQ gave final approval of the version to be submitted and any revised version. All authors contributed to the article and approved the submitted version.

## Conflict of Interest

The authors declare that the research was conducted in the absence of any commercial or financial relationships that could be construed as a potential conflict of interest.
