# Supplementary material for: Differential Host Pro-Inflammatory Response to Mycobacterial Cell Wall Lipids Regulated by the Mce1 Operon
Source: Front Immunol. 2020 Aug 18;11:1848. doi: 10.3389/fimmu.2020.01848 (PMC7461851; doi:10.3389/fimmu.2020.01848)
Supplement: Supplementary file 1 [file Data_Sheet_1.pdf]

Supplementary Material

Supplementary Table 1: List of primers sets used for qPCR screening.

Gene	Nucleotide sequences (5' to 3')
TNF-α Forward	CTACCTTGTTGCCTCCTCTTT
TNF-α Reverse	GAGCAGAGGTTCAGTGATGTAG
IL-6 Forward	CGAGAGTCCTTCAGAGAGATACA
IL-6 Reverse	CCTTCTGTGACTCCAGCTTATC
IL-1β Forward	TCGTGCTGTCGGACCCATAT
IL-1β Reverse	GTCGTTGCTTGGTTCTCCTTGT
IL-12 Forward	AAAGCAGGGCCTAGACATTC
IL-12 Reverse	CTCTGGGAAGGCCCATTTATTA
PPAR-γ Forward	CTGGCCTCCCTGATGAATAAAG
PPAR-γ Reverse	AGGCTCCATAAAGTCACCAAAG
TR4 Forward	CTGATAGCCACTCCCACATTT
TR4 Reverse	GAACTGTACCATCCTCACGTATC
RAR Forward	CTGGATCAATGCCACCTCTC
RAR Reverse	GGGACTGTGCTCTGCTATATTC
LXR-α Forward	GCTACAACCACGAGACAGAA
LXR-α Reverse	GAGAACTCGAAGATGGGATTGA
TGF-β Forward	GGTGGTATACTGAGACACCTTG
TGF-β Reverse	CCCAAGGAAAGGTAGGTGATAG
GAPDH Forward	TCAACGGCACAGTCAAGG
GAPDH Reverse	ACTCCACGACATACTCAGC
β-actin Forward	GAGGTATCCTGACCCTGAAGTA
β-actin Reverse	CACACGCAGCTCATTGTAGA

Supplementary Table 2: $2^{-\Delta\Delta CT}$ values* of RT-PCR analysis of selected cytokines and lipid-sense nuclear receptor (LSNR) genes

	**Target genes**								
**Samples**	**TNF-α**	**IL-6**	**IL-1β**	**IL-12**	**TGF-β**	**PPAR-γ**	**TR4**	**RAR**	**LXR-α**
**WT-1**		674.31	138.17	0.94	1.26	0.47	0.59	0.46	1.40
**WT-2**	7.29	1,974.16	385.28	2.67	1.81	0.87	0.85	0.71	1.96
**WT-3**	14.47	996.40	378.32	1.35	1.71	0.65	0.85	0.76	1.85
**WT-4**	6.46	1,938.90	30.32	4.79	1.48				0.90
**WT-5**	5.51	1,083.72		5.98	0.67	0.29	0.89	0.62	0.46
**WT-6**	14.69	835.08	64.85	6.15	1.05	0.52	0.64	1.00	1.24
**Δmce1-1**	2.54			1.97	3.80	0.27	1.20	1.70	2.72
**Δmce1-2**	1.80	96.59	50.20	1.20	1.54	0.48	0.84	0.84	0.95
**Δmce1-3**	6.22	32.97	50.22	1.38	1.72	0.25	1.71	1.31	1.87
**Δmce1-4**			6.74	2.86	0.59	1.19	1.38	1.00	1.03
**Δmce1-5**	4.02	33.12	8.69	3.56	0.56	2.73	1.45	1.26	1.78
**Δmce1-6**	2.34	26.84	14.43	3.29	0.35	3.78	0.96	1.35	0.83

*$2^{-\Delta\Delta CT}$ normalized to GAPDH and *β-actin*

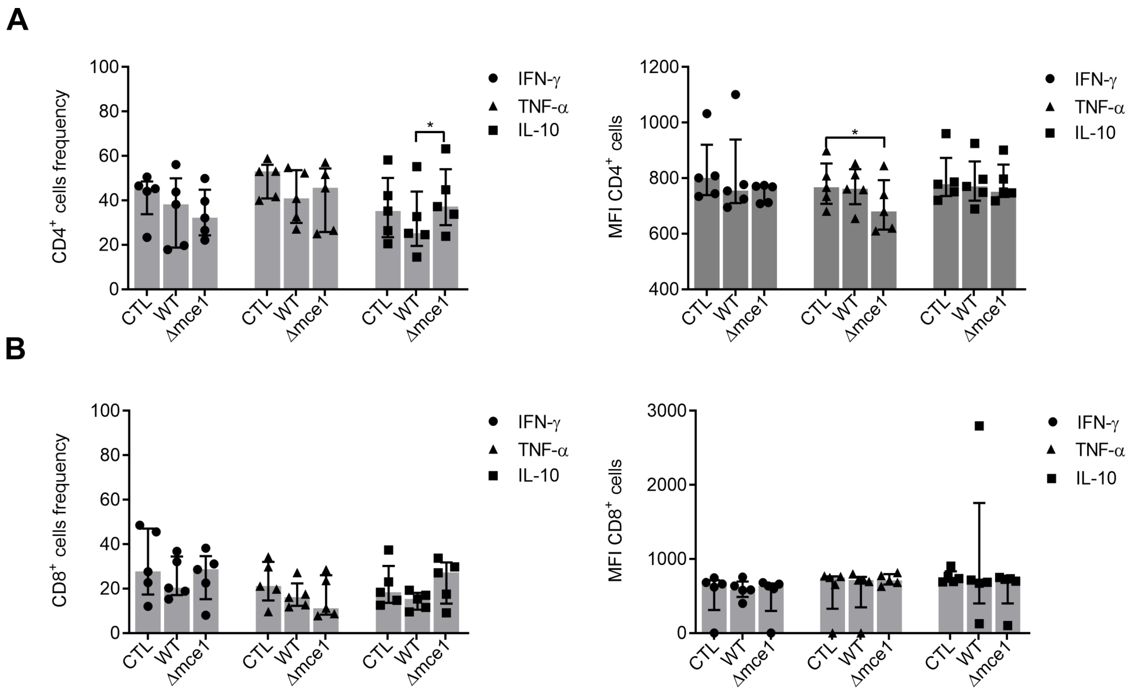

Supplementary Figure 1. IFN-γ-, TNFα- and IL-10-producing CD4^{+} and CD8^{+} T cells from six healthy donors in a co-culture assay. Non-adherent cells were added to a culture containing adherent cells exposed to apolar lipids extracted from wild type vs *mce1* operon mutant *M. tuberculosis*. (*A)*, Frequency and mean fluorescence intensity of IFN-γ-, TNF-α and IL-10 produced by CD4^{+} T cells. (*B*), Frequency and mean fluorescence intensity of IFN-γ-, TNF-α and IL-10 produced by CD8^{+} T cells; n = 5/group. Statistical significance was evaluated by one-way ANOVA test and for non-parametric samples the Kruskal–Wallis test, followed by Dunn's post-test. WT = Mtb wild type strain; Δmce1 = Mtb strain disrupted in mce1 operon; CTL = no stimulus.

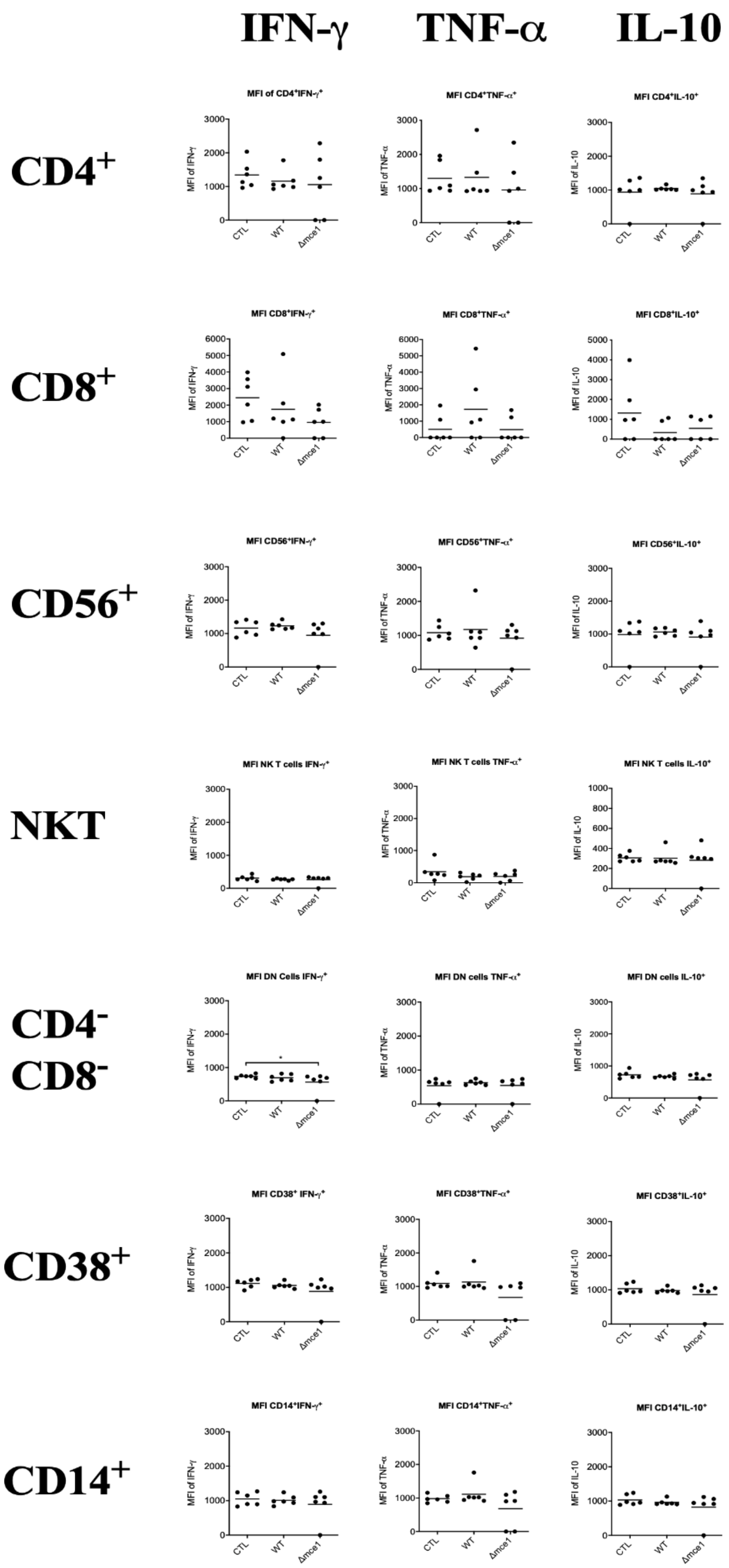
IFN-γ
TNF-α
IL-10
CD4+
CD8+
CD56+
NKT
CD4-
CD8-
CD38+
CD14+
MFI of CD4+IFN-γ+
MFI CD4+TNF-α+
MFI CD4+IL-10+
MFI CD8+IFN-γ+
MFI CD8+TNF-α+
MFI CD8+IL-10+
MFI CD56+IFN-γ+
MFI CD56+TNF-α+
MFI CD56+IL-10+
MFI NK T cells IFN-γ+
MFI NK T cells TNF-α+
MFI NK T cells IL-10+
MFI DN Cells IFN-γ+
MFI DN cells TNF-α+
MFI DN cells IL-10+
MFI CD38+ IFN-γ+
MFI CD38+TNF-α+
MFI CD38+IL-10+
MFI CD14+IFN-γ+
MFI CD14+TNF-α+
MFI CD14+IL-10+
MFI of IFN-γ
MFI of TNF-α
MFI of IL-10
CTL
WT
Δmce1

Supplementary Figure 2A. Mtb Lipid Stimulated Cell Panel. Peripheral blood mononuclear cell (PBMC) and cytokine production from six TB patients exposed to apolar lipids extracted from wild type vs *mce1* operon mutant *M. tuberculosis.* Frequency of $CD4^+$, $CD8^+$, $CD56^+$, NKT, $CD4^-CD8^-$, $CD38^+$ and $CD14^+$ cells data were represented by median ± IQR; n = 6 for each group. Statistical significance was evaluated by Mann-Whitney U test. * $p < 0.05$. WT = Mtb wild type strain; Δmce1 = Mtb strain disrupted in mce1 operon; CTL = no stimulus.

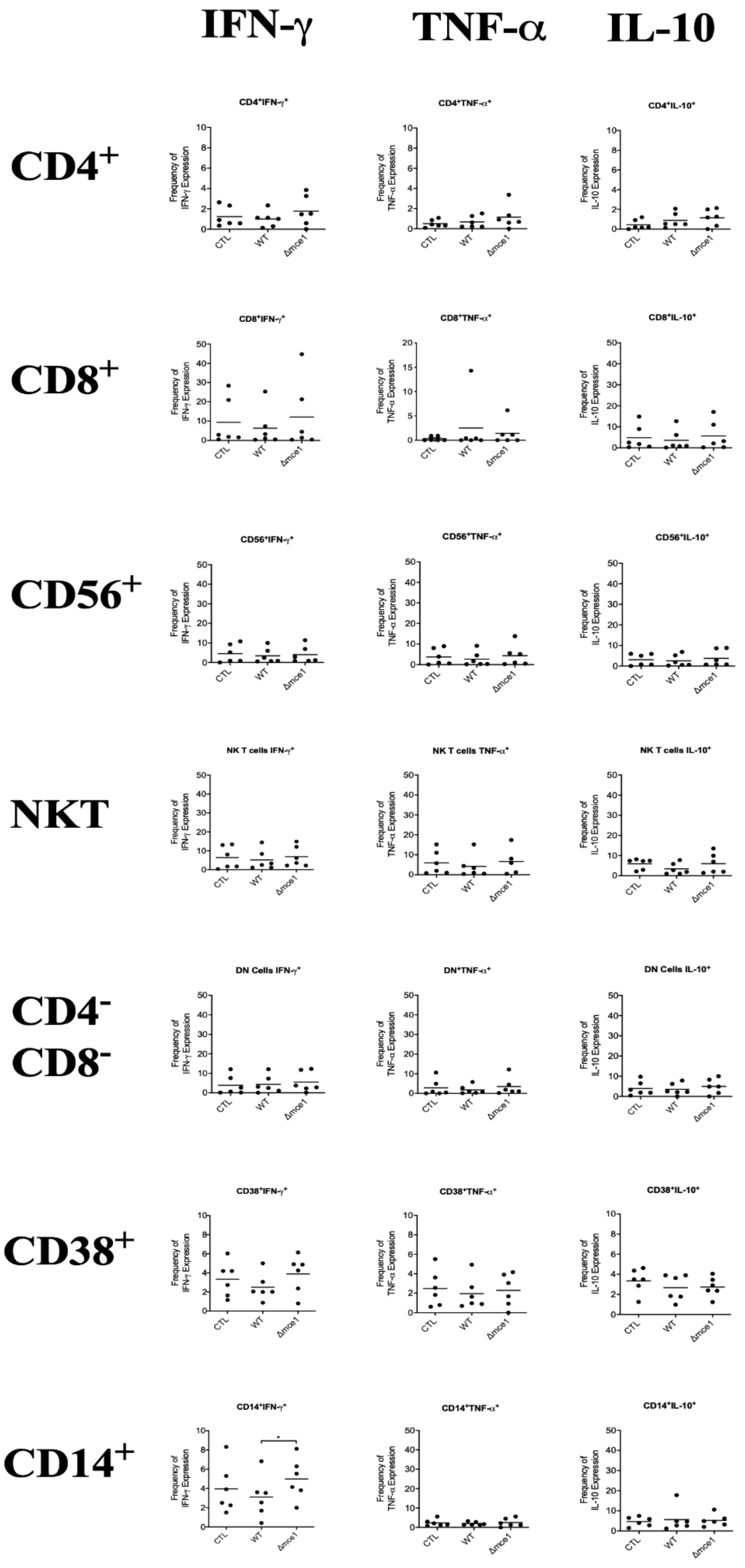
IFN-γ
TNF-α
IL-10
CD4+
CD8+
CD56+
NKT
CD4-
CD8-
CD38+
CD14+
CD4+IFN-γ+
CD4+TNF-α+
CD4+IL-10+
CD8+IFN-γ+
CD8+TNF-α+
CD8+IL-10+
CD56+IFN-γ+
CD56+TNF-α+
CD56+IL-10+
NK T cells IFN-γ+
NK T cells TNF-α+
NK T cells IL-10+
DN Cells IFN-γ+
DN+TNF-α+
DN Cells IL-10+
CD38+IFN-γ+
CD38+TNF-α+
CD38+IL-10+
CD14+IFN-γ+
CD14+TNF-α+
CD14+IL-10+
Frequency of IFN-γ Expression
Frequency of TNF-α Expression
Frequency of IL-10 Expression
CTL
WT
Δmce1
*

Supplementary Figure 2B. Mtb Lipid Stimulated Cell Panel. Peripheral blood mononuclear cell (PBMC) and cytokine production from six TB patients exposed to apolar lipids extracted from wild type vs *mce1* operon mutant *M. tuberculosis*. Quantification of the mean fluorescence intensity of $CD4^+$, $CD8^+$, $CD56^+$, NKT, $CD4^-$ $CD8^-$, $CD38^+$ and $CD14^+$ cells data were represented by median ± IQR; n = 6 for each group. Statistical significance was evaluated by Mann-Whitney U test. * $p < 0.05$. WT = Mtb wild type strain; Δmce1 = Mtb strain disrupted in *mce1 operon*; CTL = no stimulus.
